# Maternal and child factors associated with early initiation of breastfeeding in Chad: evidence from nationally representative cross-sectional data

**DOI:** 10.1093/inthealth/ihab060

**Published:** 2021-10-06

**Authors:** Bright Opoku Ahinkorah, Abdul-Aziz Seidu, Eugene Budu, Aliu Mohammed, Collins Adu, Edward Kwabena Ameyaw, Kwaku Kissah-Korsah, Faustina Adoboi, Sanni Yaya

**Affiliations:** School of Public Health, Faculty of Health, University of Technology Sydney, Australia; Department of Population and Health, University of Cape Coast, Cape Coast, Ghana; College of Public Health, Medical and Veterinary Sciences, James Cook University, Townsville, Queensland, Australia; Department of Population and Health, University of Cape Coast, Cape Coast, Ghana; Department of Health, Physical Education and Recreation, University of Cape Coast, Cape Coast, Ghana; Department of Health Promotion, Education and Disability Studies, Kwame Nkrumah University of Science and Technology, Kumasi, Ghana; School of Public Health, Faculty of Health, University of Technology Sydney, Australia; Department of Population and Health, University of Cape Coast, Cape Coast, Ghana; Cape Coast Nursing and Midwifery Training College, Cape Coast, Ghana; University of Parakou, Faculty of Medicine, Parakou, Benin

**Keywords:** Chad, child health, early initiation of breastfeeding, global health, mother, newborn

## Abstract

**Background:**

Early initiation of breastfeeding (EIB) is an inexpensive practice but has a substantial potential to reduce neonatal morbidity. Therefore, this study investigated the maternal and child-related factors associated with EIB and makes recommendations that could help improve the practice in Chad.

**Methods:**

We used data from the children's recode file of the 2014–2015 Chad Demographic and Health Survey. A total of 3991 women ages 15–49 y who had last-born children in the 2 y preceding the survey were included in our study. The outcome variable for the study was EIB. Both descriptive (frequencies and percentages) and inferential (binary logistic regression) analyses were carried out. All results of the binary logistic analyses are presented as adjusted odds ratios (aORs) with 95% confidence intervals (CIs).

**Results:**

We found the prevalence of EIB in Chad to be 23.8%. In terms of maternal factors, the likelihood of EIB was high among non-working women (aOR 1.37 [95% CI 1.18 to 1.59]), the richest wealth quintile women (aOR 1.37 [95% CI 1.04 to 1.79]) and non-media-exposed women (aOR 1.58 [95% CI 1.24 to 2.02]) compared with working women, the poorest wealth quintile women and media-exposed women, respectively. EIB was lower among children whose mothers had one to three antenatal care visits (ANC; aOR 0.73 [95% CI 0.61 to 0.87]) and four or more ANC visits (aOR 0.80 [95% CI 0.66 to 0.97]) compared with those who had no ANC visits. With the child factors, EIB was higher among mothers of children who were smaller than average size at birth compared with those of larger than average birth size (aOR 1.47 [95% CI 1.24 to 1.74]). Mothers of children of fifth-order or more births compared with those of first-order births (aOR 1.51 [95% CI 1.07 to 2.12]) and those who were delivered through vaginal birth compared with those delivered through caesarean section (aOR 4.71 [95% CI 1.36 to 16.24]) were more likely to practice EIB.

**Conclusions:**

Maternal and child-related factors play roles in EIB in Chad. Hence, it is important to consider these factors in maternal and neonatal health interventions. Such initiatives, including training of outreach health workers, health education, counselling sessions and awareness-raising activities on breastfeeding geared towards EIB should be undertaken. These should take into consideration the employment status, wealth quintile, exposure to mass media, size of the baby at birth, ANC visits, parity and delivery method.

## Introduction

Globally, 2.5 million newborns died in their first month of life in 2018, representing 47% of all mortalities in children <5 y of age.^1^ Thus, approximately 7000 neonatal deaths were recorded daily across the world. Although there had been a steady decline in neonatal mortality across the globe, sub-Saharan Africa (SSA) continues to have the highest neonatal mortality rate of 28 deaths per 1000 live births as of 2018.^1^ According to the United Nations Inter-agency Group for Child Mortality Estimation, Chad has one of the highest neonatal mortality rates (34 deaths per 1000 live births) in SSA.^2^ Most newborn deaths occur from conditions associated with a lack of quality care at birth or treatment immediately after birth.^1^ Thus these deaths generally occur from preventable causes, and early initiation of breastfeeding (EIB), which is usually defined as breastfeeding within 1 h of birth, is one of the recommended immediate newborn care practices that reduce the risk of neonatal deaths.^3–5^

EIB is an inexpensive practice but has a substantial potential to reduce neonatal morbidity and mortality.^6^ Meta-analysis studies have shown that EIB is associated with a decreased risk of neonatal mortality while later initiation is associated with increased risk.^4,5^ The practice was also found to significantly reduce the risk of severe illnesses in early newborns^7^ and the risk of postpartum haemorrhage and its associated maternal deaths.^4^ Thus early EIB could improve both neonatal and maternal health outcomes.

Despite these benefits, available data suggest that <50% of newborns are breastfed within 1 h of birth in SSA.^3^ The proportions differ from one country to another, with Chad being one of the countries with the lowest percentage (34%) of EIB, surpassing only Guinea (17%), Ivory Coast (31%), Nigeria (33%) and Gabon (33%) among 33 sub-Saharan Africa countries.^3^ Despite recommendations for mothers to practice EIB, the rate has not seen much improvement in Chad.

Several reasons have been given for delayed initiation of breastfeeding in Africa. These include the belief that breast milk does not flow immediately after birth,^8–11^ the belief that colostrum is harmful or not healthy for babies,^8,9,12,13^ the perception that the mother and baby must have a bath after delivery,^9,10^ the belief that babies sleep and do not show any sign of hunger after deleivery^10,14^ and the perceived need for mother and baby to rest after delivery.^12,13^ Other factors such as delivery by caesarean section, being unmarried and exposure to infant formula advertisements have also been associated with delayed initiation of breastfeeding.^15^ In contrast, facilitators of EIB include frequent antenatal care (ANC) visits, receiving education on EIB,^16^ birthing at a health facility,^10^ living in an urban area,^17^ multiparity,^17,18^ having a large baby and being unemployed.^17^

Studies on EIB in SSA have largely focused on sociocultural factors associated with EIB. Very little is known about individual (maternal or child-related) factors associated with EIB in Chad, thus identifying maternal and child-related factors in EIB is key in developing feasible strategies that could help improve the practice among breastfeeding mothers. Therefore, this study investigated the maternal and child-related factors associated with EIB and makes recommendations that could help improve the practice in Chad.

## Methods

### Data source

We used data from the 2014–2015 Chad Demographic and Health Survey (DHS). Specifically, we used data from the children's recode file, which contains data on births that occurred in the past 5 y. The DHS is a nationally representative survey that is conducted in >85 low- and middle-income countries globally. It focuses on essential maternal and child health markers, including breastfeeding practices.^19^ The survey employs a two-stage stratified sampling technique, which makes the data nationally representative. The study by Aliaga and Ruilin^20^ provides details of the sampling process. A total of 3991 women ages 15–49 y who had a last-born child in the 2 y preceding the survey (4217) and practiced breastfeeding (3991) were included in our study. Hence, the actual sample size used for this study was 3991. We relied on the Strengthening the Reporting of Observational Studies in Epidemiology statement in conducting this study and writing the manuscript.

## Definition of variables

### Outcome variable

The outcome variable for the study was EIB. It was derived from the question, ‘How long after birth did you first put (NAME) to the breast?’ The responses were immediately, hours and days. The responses were then dichotomized as early initiation of breastfeeding=1, if women reported immediately or within the first hour of breastfeeding, and later initiation=0, if women reported otherwise. The derivation and categorization of this variable was based on the literature.^21–23^

### Explanatory variables

The study used 15 explanatory variables. These variables were considered principally because of their statistically significant relationship with EIB in previous studies.^21–25^ These variables were grouped into maternal and child factors. The maternal factors included mother's age, marital status, employment status, frequency of reading a newspaper, frequency of listening to radio, frequency of watching television, place of residence, number of ANC visits and wealth quintile. In the DHS, the wealth quintile is computed using different household ownerships and characteristics following the principal component analysis technique.^19^ Frequency of reading a newspaper, frequency of listening to radio and frequency of watching television were originally categorized into not at all, less than once a week and at least once a week. These were recoded as ‘yes’ (less than once a week and at least once a week) and ‘no’ (not at all). Finally, exposure to media was generated and categorized as ‘not exposed’ and ‘exposed’. ‘Not exposed’ represented three ‘no’ answers for all the media sources, while ‘exposed’ represented at least one ‘yes’ for all the media sources. The child factors used for the study were birth order, type of delivery assistance, place child was delivered, type of delivery, twin status of the child and mother's subjective self-report of the size of the child at birth. The data collection team determined the size of the child at birth based on the description given by the mother.

### Statistical analyses

The data were analysed with Stata version 14.0 (StataCorp, College Station, TX, USA). The analyses were done in three steps. The first step was the computation of the prevalence of EIB in Chad. The second step was a bivariate analysis involving calculation of the proportion of EIB across the maternal and child factors with their significance levels using the χ^2^ test of independence. To check for a high correlation among the explanatory variables, a test for multicollinearity was carried out using the variance inflation factor (VIF) and the results showed no evidence of high collinearity (mean VIF=2.12, maximum VIF=6.06, minimum VIF=1.02). Afterwards, two binary logistic regression models were built. Only variables that were significant (p<0.05) from the second step were included in the binary logistic regression (see Table 2). Model I constituted a bivariate analysis between the maternal factors and EIB. We added child factors to the initial model in model II. All frequency distributions were weighted and the survey command (svy) in Stata was used to adjust for the complex sampling structure of the data in the regression analyses. The results of the logistic analyses were presented as adjusted odds ratios (aORs) with 95% confidence intervals (CIs).

### Ethical approval

This was a secondary analysis of data and therefore no further approval was required, as the data are available in the public domain. Further information about Chad DHS data usage and ethical standards are available at https://dhsprogram.com/what-we-do/survey/survey-display-465.cfm.

## Results

### Description of participants

Of the 3991 participants, the modal age was 25–29  y (27.6%). The majority of the participants lived in rural areas (81.4%), were married (85.5%), had no formal education (62.4%) and were not exposed to media (84.5%). The modal wealth quintile was poorer (22.1%) and the model number of ANC visits was one to three (33.9%). The modal size of the child at birth was larger than average (48.8%) and birth order was fifth or more (43.4%). The majority of the children were delivered at home (76.1%), were assisted by traditional birth attendance (74.0%), were delivered through vaginal delivery (98.7%) and were not twins (98.4%).

### Proportion of EIB in Chad

**Figure 1.**
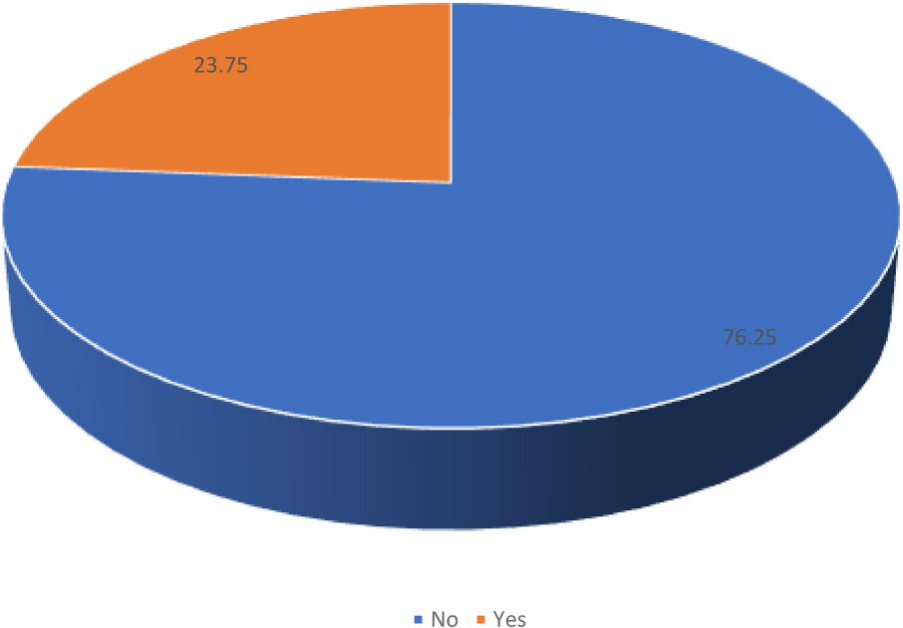
Proportion of EIB in Chad.

The proportion of mothers who initiated breastfeeding early was 23.8% (Figure 1).

### Bivariate results on the determinants of EIB in Chad

Table 1 presents results of the maternal and child factors and EIB in Chad. Apart from place of residence and twin status, all the explanatory variables had significant associations with EIB. In terms of the distribution of the significant variables with EIB, the highest prevalence of EIB was among women ages 40–44 y (29.0%), those who were married (24.6%), women with no education (26.9%), those who were not working (27.7%), those in the richer wealth quintile (26.9%), women who were not exposed to media (25.1%) and women who had no ANC visits (28.8%). For the child factors, women with smaller than average children (29.4%), those who had a birth order of five or more (26.9%), those who delivered at home (24.0%), those who had the assistance of a traditional birth attendant during delivery (24.3%) and those who had a vaginal delivery (24.0%) had the highest prevalence of EIB.

**Table 1.** Distribution of EIB in Chad by explanatory variables (weighted N=3991)

Variables	Weighted N	Weighted %	EIB (%)	p-Value
Maternal factors				
Age (years)				0.047
15–19	563	14.1	19.8	
20–24	927	23.2	20.1	
25–29	1102	27.6	25.2	
30–34	746	18.7	27.8	
35–39	454	11.4	23.5	
40–44	155	3.9	29.0	
45–49	44	1.1	28.8	
Residence				0.10
Urban	741	18.6	23.0	
Rural	3250	81.4	23.9	
Marital status				0.001
Never married	52	1.3	16.2	
Married	3410	85.5	24.6	
Cohabiting	365	9.2	17.1	
Widowed/divorced/separated	164	4.1	23.9	
Education				<0.001
No education	2491	62.4	26.9	
Primary	1021	25.6	18.0	
Secondary/higher	479	12.0	19.4	
Employment				<0.001
Not working	1899	47.6	27.7	
Working	2092	52.4	20.1	
Wealth quintile				0.001
Poorest	878	22.0	22.8	
Poorer	884	22.1	20.5	
Middle	821	20.6	23.5	
Richer	770	19.3	26.9	
Richest	638	16.0	26.1	
Exposure to mass media^a^				<0.001
Not exposed	3373	84.5	25.1	
Exposed	618	15.5	16.7	
ANC visits				<0.001
None	1326	33.2	28.8	
1–3	1354	33.9	21.4	
≥4	1311	32.9	21.1	
Child factors				
Size of child at birth				<0.001
Larger than average	1949	48.8	21.4	
Average	1063	26.6	22.9	
Smaller than average	979	24.5	29.4	
Birth order				<0.001
1	634	15.9	17.2	
2–4	1625	40.7	22.9	
≥5	1732	43.4	26.9	
Place of delivery				0.006
Home	3038	76.1	24.0	
Health facility	953	23.9	23.0	
Assistant during delivery				0.002
Traditional birth attendance	2953	74.0	24.3	
Skilled birth attendance	1038	26.0	22.2	
Type of delivery				0.002
Vaginal birth	3939	98.7	24.0	
Caesarean section	52	1.3	4.7	
Birth status				0.818
Single birth	3927	98.4	23.7	
Multiple birth	64	1.6	26.9	

Source: 2014–2015 Chad DHS.

a Exposure to media refers to exposure to radio, television or newspapers.

### Results of multivariable logistic regression analysis

Table 2 presents results of the binary logistic regression analysis on the maternal and child factors associated with EIB in Chad. Model II, which is the complete model, presents the results for maternal factors associated with EIB while controlling for child factors.

**Table 2.** Multivariable logistic regression analysis on determinants of EIB in Chad

Variables	Model I, aOR (95% CI)	Model II, aOR (95% CI)
Maternal factors		
Age (years)		
15–19	1	1
20–24	0.98 (0.76 to 1.26)	0.82 (0.62 to 1.09)
25–29	1.18 (0.93 to 1.50)	0.94 (0.69 to 1.28)
30–34	1.34* (1.04 to 1.73)	1.06 (0.75 to 1.49)
35–39	1.06 (0.79 to 1.43)	0.82 (0.56 to 1.20)
40–44	1.09 (0.73 to 1.64)	0.82 (0.51 to 1.33)
45–49	1.45 (0.74 to 2.81)	1.12 (0.55 to 2.29)
Marital status		
Never married	1	1
Married	1.21 (0.53 to 2.72)	1.02 (0.44 to 2.38)
Cohabiting	0.88 (0.37 to 2.10)	0.82 (0.33 to 2.00)
Widowed/divorced/separated	3.99* (1.31 to 12.16)	3.65* (1.18 to 11.30)
Education		
None	1.24 (0.94 to 1.63)	1.08 (0.81 to 1.43)
Primary	0.96 (0.71 to 1.31)	0.88 (0.64 to 1.20)
Secondary/higher	1	1
Employment		
Not working	1.36*** (1.17 to 1.58)	1.37*** (1.18 to 1.59)
Working	1	1
Wealth quintile		
Poorest	1	1
Poorer	0.90 (0.72 to 1.14)	0.89 (0.71 to 1.13)
Middle	1.24 (1.00 to 1.56)	1.23 (0.98 to 1.54)
Richer	1.27* (1.02 to 1.59)	1.28* (1.02 to 1.70)
Richest	1.37* (1.06 to 1.77)	1.37* (1.04 to 1.80)
Exposure to mass media^a^		
Not exposed	1.60*** (1.26 to 2.04)	1.58*** (1.24 to 2.02)
Exposed	1	1
ANC visits		
None	1	1
1–3	0.72*** (0.60 to 0.86)	0.73*** (0.61 to 0.87)
≥4	0.77** (0.64 to 0.93)	0.80* (0.66 to 0.97)
Child factors		
Size of child at birth		
Larger than average		1
Average		0.92 (0.77 to 1.11)
Smaller than average		1.47*** (1.24 to 1.74)
Birth order		
1		1
2–4		1.44* (1.09 to 1.91)
≥5		1.51* (1.07 to 2.12)
Place of delivery		
Home		0.89 (0.57 to 1.38)
Health facility		1
Assistant during delivery		
Traditional birth attendance		1.14 (0.74 to 1.75)
Skilled birth attendance		1
Type of delivery		
Vaginal birth		4.71* (1.36 to 16.24)
Caesarean section		1
Pseudo R^2^	0.028	0.039
N	3991	3991

Source: 2014–2015 Chad DHS.

*p<0.05, **p<0.01, ***p<0.001; 1, reference category.

a Exposure to media refers to exposure to radio, television or newspapers.

### Maternal factors associated with early initiation of breastfeeding in Chad

In terms of the maternal factors, the likelihood of EIB was higher among non-working working women (aOR 1.37 [95% CI 1.18 to 1.59]), the richest wealth quintile women (aOR 1.37 [95% CI 1.04 to 1.80]) and non-media-exposed women (aOR 1.58 [95% CI 1.24 to 2.02]) compared with working women, the poorest wealth quintile women and media-exposed women, respectively. EIB was lower among children whose mothers had one to three ANC visits (aOR 0.73 [95% CI 0.61 to 0.87]) and four or more ANC visits (aOR 0.80 [95% CI 0.66 to 0.97]) compared with those who had no ANC visits.

### Child factors associated with early initiation of breastfeeding in Chad

With the child factors, EIB was higher among mothers of children who were smaller than average at birth compared with those who were larger than average birth size (aOR 1.47 [95% CI 1.24 to 1.74]). Mothers of children of birth order five or more compared with those of first-order births (aOR 1.51 [95% CI 1.07 to 2.12]) and those who were delivered through vaginal birth compared with those delivered through Caesarean section (aOR 4.71 [95% CI 1.36 to 16.24]) were more likely to practice EIB.

## Discussion

EIB is a simple, cost-effective and lifesaving intervention for the health of newborns.^26^ This cross-sectional study assessed the maternal and child-related factors associated with EIB in Chad. In this study, the prevalence of EIB in Chad was found to be 23.8%, which is lower compared with the prevalence of previous studies in Ethiopia (74.1%),^27^ Zimbabwe (58.3%),^25^ India (36.4%)^28^ and Bangladesh (24%)^29^ but was higher than in Pakistan (8.5%).^30^ The mother's employment status, wealth quintile, exposure to media, ANC visits and marital status were found to be significantly associated with EIB, while the size of the baby at birth, type of delivery and birth order were associated with EIB in terms of child factors.

Our study revealed that maternal employment status has a significant association with EIB, with mothers who were not working being more likely to initiate breastfeeding early than mothers who were working. This finding is consistent with previous studies from Namibia^31^ and Nigeria.^17^ However, our results showed that the richest mothers were more likely to initiate breastfeeding early than the poorest mothers. This could be due to several reasons, including better access to and availability of health resources. A similar finding has been reported in Nigeria.^17^ However, this contradicts the finding from a study in Namibia^32^ that found EIB is higher among mothers in households with a poor wealth index as compared with the rich households.

Findings from this study show that EIB is lower among women who were exposed to mass media (radio, television and newspapers) as compared with women who were not exposed to mass media. This finding contradicts previous studies^33,34^ that reported mothers who frequently read newspapers or listen to radio or watch television were more likely to initiate early breastfeeding. Exposure to other information sources relating to breastfeeding practices during the period of ANC and postnatal care can also play an important role in encouraging mothers to initiate early breastfeeding.^35^ In terms of the association between mass media exposure and EIB, this could be that information on mass media did not capture breastfeeding messages. Promotional messages on breastfeeding practices should continue to sustain the practice of EIB in the country.

Furthermore, women with one to three and four or more ANC visits were less likely to practice EIB compared with those who had no ANC visits. These results contradict previous studies conducted in West African states,^35–37^ India^24^ and Nepal.^38^ The Baby-Friendly Hospital Initiative provides health messages and breastfeeding counselling that encourage the practice of EIB.^35^ Evidence from the literature^39,40^ shows that breastfeeding counselling during ANC visits is positively associated with mothers’ adherence to the World Health Organization EIB practices. Hence supporting and promoting ANC visits in Chad could produce remarkable improvements in EIB practices.

Women who were widowed, divorced or separated were more likely to practice EIB compared with those who were never married. This finding contradicts the finding of a previous study conducted in Namibia^31^ that showed EIB was more likely among married mothers. The possible reason could be the psychosocial support from their partners.

Also, findings from Nigeria^17,41^ and Brazil^42^ showed that EIB was more likely among mothers who had large babies at birth. This contradicts the finding of the present study that EIB was more likely among mothers who had smaller than average babies at birth. It is argued that large babies are healthy and capable of suckling and therefore lead to EIB.^42^ However, it is thought that small babies need more care and breastfeeding immediately after birth to support and maintain their body temperature and facilitate proper weight gain after birth.^32^ Again, we observed that women who had vaginal delivery were more likely to practice EIB as compared with women who delivered through caesarean section. The result is consistent with studies in Ethiopia,^43^ Uganda,^44^ Saudi Arabia^45^ and Bangladesh^46^ that also revealed mothers who had a vaginal delivery commenced early breastfeeding. The delay in initiation of breastfeeding could be due to the condition of mothers and critical conditions of newborns after caesarean delivery.^47^ Since caesarean delivery is becoming an increasingly common mode of delivery, providing services that inform mothers about the relevance of EIB for newborns and themselves is needed.

Mothers of children with a birth order of two to four and five or more were more likely to practice EIB compared with those of first-order children. This finding is consistent with a previous study conducted in the Economic Community of West African States.^35^ The plausible reason could be the poor use of maternal health services by first-time mothers and inexperience related to breastfeeding of first-time mothers. Also, delivery complications are more likely during the first pregnancy, which may end up in separation of the mother from the child and can delay EIB.^18^

### Strengths and limitations

A strength of this study lies in the use of a nationally representative dataset to examine both maternal and child-related factors associated with EIB in Chad. Also, the probability method employed in selecting survey respondents matched with appropriate analytical procedure makes the results of the study robust. However, our results should be interpreted with caution. First, causality cannot be established due to the cross-sectional nature of the study. Second, most of the variables are from maternal self-reports, so there may be an accuracy issue. Recall bias may occur, resulting in inaccurate responses due to the time interval between delivery and the interview. Third, the DHS is prone to incomplete or partial reporting of responses. Additionally, complex questionnaires may inevitably allow for inconsistent responses to be recorded for different questions. Finally, the timing of the survey questions differs. For instance, while questions on the child factors and initiation of breastfeeding were in reference to events that occurred at the birth of the last child, the variables on maternal factors were gathered at the time of the survey.

## Conclusions

Maternal and child-related factors play roles in EIB in Chad, thus it is important to consider these factors in maternal and neonatal health interventions. To boost EIB, interventions must be implemented during pregnancy and after childbirth. Such interventions should include training of outreach health workers, health education, counselling sessions and awareness-raising activities on breastfeeding geared towards EIB both during pregnancy and after birth. These should take into consideration the marital status, mother’s age, size of the baby at birth, parity, employment status of the mother, educational level of the mother, exposure to mass media, wealth index and place of residence. Furthermore, to encourage EIB, caesarean section policies should focus on less separation of mothers and their babies after surgery. To promote EIB, health education should include communications promoting EIB.

## Data Availability

Data for this study were sourced from the Chad Demographic and Health Survey and are available from https://dhsprogram.com/data/available-datasets.cfm.
